# A New Light on Vitamines

**Published:** 1921-06-25

**Authors:** 


### DEFICIENCY DISEASES.

### A New Light on Vitamines.

The most recently published work of Dr. Robert McCarrison* appears as a volume which should be carefully read by every medical man interested in the wider health problems. Deficiency diseases, by which we mean pathological conditions arising from a deficiency of vitamines in the diet, have lately claimed a great deal of both professional and lay attention. At the same time, the idea that the absence of a small but indispensable ingredient of diet can have such far-reaching results is, by its very simplicity, liable to obscure the main issues We are in many ways beginning to realise that disease is not only produced by actively destructive agents, but also that a negative influence, or deficiency, can be equally potent in this respect. It is intensely interesting, therefore, to follow with this author some of the finer ramifications of the experimental work which the new idea involves.

### A Definition.

It is hard to find a more apt definition of vitamine action than that provided by the author. It has the advantage of including a simile comprehensible to most of us.

" Vitamines are as a spark which ignites the fuel mixture of a petrol-driven engine, liberating its energy. The spark is of no use without the fuel, nor the fuel without the spark—nay more, the efficiency of the spark is dependent in great measure on the composition of the fuel mixture."

The parallel between the vitamine and the electric spark is extraordinarily perfect. The end result of absence either of food or of the essential vitamine is the same. In the first case there is direct starvation; in the second, though food be taken in abundance, it is not utilised. There is indirect starvation. Dr. McCarrison's earlier researches have shown, with a high degree of completeness, that although absence of the now differentiated vitamines, named with their solubilities A, B, and C, tends to produce certain definite pathological complexes, *e.g.*, rickets, scurvy, etc., yet underlying all of them are those degenerative changes brought about by direct starvation. The more one reads of this and others of the author's books the more completely can one realise that between these different deficiency complexes, no hard-and-fast line can be drawn. The predominant absence of one or other of our vitamines, as at present classified, may tend to produce a predominant symptom, but in each instance the phenomena of true starvation are also strongly exemplified. In any case, it must be fully understood that in real life we can by no means pin down the deficiency diseases as falling naturally into separate and definite terminological compartments. Let us again quote Dr. McCarrison's introduction. "The effects on the nervous system of a dietary devoid of vitamines and disproportionately rich in starch, as observed on animals, have often been emphasised to the almost complete exclusion of other equally important, if less prominent, symptoms. Long before the nervous symptoms supervene, others, such as loss of appetite, impaired digestion, diarrhœa, colitis, unhealthy skin, low temperature, slow respiration, cardio-vascular depression, progressive anæmia and asthenia, result from deficient and ill-balanced food. Do not these form a disease syndrome which is, in children, as familiar as its cause is unrecognised? "

* " Studies in Deficiency Disease," by Robert McCarrison, M.D., D.Sc. (London: Henry Frowde and Hodder & Stoughton. Price 30s.)

### Predisposition and Protection.

With so insidious an onset as deficiency disease must always have, it is of particular interest to note the protective instincts seen in the animal kingdom. It is an invariable rule that experimental animals fed on " deficient " diets, will, when the opportunity occurs, always first select articles likely to make good the deficiency. Monkeys, in these circumstances, will spend much time endeavouring to capture flies, which they eagerly consume. Even in the large open cages of the Zoo, they will rush to secure a fragment of fresh vegetable. Fowls will consume their own feathers and those of their fellows. If captive animals are given a choice of food such as biscuits, some made from deficient, others from an adequate mixture, they will instinctively choose the latter. The natural desire for fresh food is universal in the animal kingdom.

But Species and Race have a marked influence upon the degree and type of disease which any deficiency apparently makes. Laboratory animals give clear examples of this. Fowls and pigeons are pre-eminently susceptible to a lack of vitamine B. Rats are far more affected by lack of A than of C. In the case of humans the variations appear to be even finer. Darling records that the same deficient diet which, in a negro of Cape Colony, produces only the mildest symptoms, will result in severe scurvy in a native of Tropical Africa. It is also stated that in a certain tin-mine in the East Indies the Chinese coolie is striken with beri-beri, while his European overseer develops sprue! This, although there is no essential difference between the conditions of their diet or daily life. Age, too, has a retarding influence upon development of deficiency symptoms. A variety of examples can be drawn to show the influence of sex, and here some conditions are more readily produced in the male, others in the female. Dr. McCarrison discusses a host of other factors; individual idiosyncrasy, imperfect balance of food, fatigue, cold, damp, panic, fear, mental depression, overcrowding, imperfect hygiene, and most important of all, the intervention of independent infectious diseases. He fully illustrates the fact that no two individuals will necessarily, with respect to symptoms and time of their onset, exhibit precisely the same response to vitamine deficiency. For the physician, especially important is it to realise that persons receiving too little vitamine are living in a state of potential morbidity which may be converted into one of actual disease by any of the factors which tend further to exhaust their health and metabolism.

THE SELECTION OF FOOD.

In the actual treatment of deficiency cases outside hospital practice, most will agree as to the difficulty of obtaining anything like a true "dietetic history." Yet a reasonably correct record of the past is essential. Even when the food *eaten* is ascertained it still remains to consider it for five separate points of view. These the author classifies as, (*a*) deficiency in vitamines, (*b*) deficiency of good biological value, (*c*) deficiency in inorganic salts, (*c*) excess of carbohydrates, and (*d*) excess of fats. It is essential to realise that although the range and variety of foods available to the patient may be wide, yet his actual diet may be faulty in any of the above respects. Dr. McCarrison gives several remarkable instances of this, drawn from his own practice and, to all engaged in health work, this final chapter forms highly instructive reading. It is impossible to summarise these cases, for it is only by their careful study that the practitioner will see how the realisation of vitamines and their existence can best be intelligently applied in everyday medicine. Dr. McCarrison's present book does not pretend to deal exhaustively with the whole subject. It contains, according to its title and in fact, a record of careful studies in deficiency disease. We heartily recommend it to our readers.

